# Abundance distributions for tree species in Great Britain: A two‐stage approach to modeling abundance using species distribution modeling and random forest

**DOI:** 10.1002/ece3.2661

**Published:** 2017-01-22

**Authors:** Louise Hill, Andy Hector, Gabriel Hemery, Simon Smart, Matteo Tanadini, Nick Brown

**Affiliations:** ^1^ Department of Plant SciencesUniversity of OxfordOxfordUK; ^2^ Sylva FoundationSylva Wood CentreLong WittenhamOxfordshireUK; ^3^ Centre for Ecology & HydrologyLancaster Environment CentreBailriggLancasterUK

**Keywords:** abundance distributions, abundance–occupancy relationships, biotic effects, mapping

## Abstract

High‐quality abundance data are expensive and time‐consuming to collect and often highly limited in availability. Nonetheless, accurate, high‐resolution abundance distributions are essential for many ecological applications ranging from species conservation to epidemiology. Producing models that can predict abundance well, with good resolution over large areas, has therefore been an important aim in ecology, but poses considerable challenges. We present a two‐stage approach to modeling abundance, combining two established techniques. First, we produce ensemble species distribution models (SDMs) of trees in Great Britain at a fine resolution, using much more common presence–absence data and key environmental variables. We then use random forest regression to predict abundance by linking the results of the SDMs to a much smaller amount of abundance data. We show that this method performs well in predicting the abundance of 20 of 25 tested British tree species, a group that is generally considered challenging for modeling distributions due to the strong influence of human activities. Maps of predicted tree abundance for the whole of Great Britain are provided at 1 km^2^ resolution. Abundance maps have a far wider variety of applications than presence‐only maps, and these maps should allow improvements to aspects of woodland management and conservation including analysis of habitats and ecosystem functioning, epidemiology, and disease management, providing a useful contribution to the protection of British trees. We also provide complete R scripts to facilitate application of the approach to other scenarios.

## 1 Introduction

Robust information on the distribution and abundance of species is essential for many applications in ecology and conservation. Advances in species distribution modeling have driven an explosion in the use of these and similar techniques, which are now widespread (Araújo & Guisan, 2006). However, the most commonly desired output from these techniques, an estimate of the probability of species occurrence, is restricted in its uses. Furthermore, due to limitations in the quality of available data, the actual output of species distribution models (SDMs) is often even less useful, producing only a relative, not absolute, likelihood of presence, and sometimes worse (Guillera‐Arroita et al., 2015; Pearce & Boyce, 2006). For many ecological questions, estimates of abundance would be far more valuable as they provide much more information about the state of populations and properties of ecosystems (Hui et al., 2009; Pearce & Ferrier, 2001; Sagarin, Gaines, & Gaylord, 2006).

Predicting abundance distributions with accuracy is challenging. Even where presence–absence or presence‐only data are easy to find, large amounts of abundance data are rarely available (Nielsen et al. 2005; Van Couwenberghe, Collet, Pierrat, Verheyen, & Gégout, 2013). Therefore, finding effective ways to model abundance is an important area of research in ecology. A variety of different approaches, including looking for a fundamental relationship between the area of occupancy and abundance (Gaston et al., 2000) and looking at how occupancy patterns change with different grain size (Hui et al., 2009) among others (Hwang & He, 2011; Wenger & Freeman, 2008), have been attempted. However, none of these has produced consistently satisfactory results and each has significant theoretical or practical limitations.

Another approach has been to investigate relationships between likelihood of occurrence and abundance. This approach assumes that species abundance and occurrence are controlled by the same or related environmental factors (Brown, 1984; Van Couwenberghe et al., 2013). Various studies have attempted to correlate the results of SDMs or related models with abundance data to produce models predicting abundance in unknown areas (Johnson & Seip, 2008; Nielsen et al. 2005; Van Couwenberghe et al., 2013). However, wide variation in the relationships between species occurrence, and abundance has been reported, with various studies showing weak relationships (Gaston et al., 2000; Nielsen et al. 2005; Van Couwenberghe et al., 2013). Another study by Pagel et al. (2014) used a hierarchical model to predict abundances in time and space using a combination of plentiful occurrence data and restricted abundance data. Their method produced unbiased results, but with very low precision in predictions, perhaps due to inflexibility in their models or not using environmental covariates. These studies have all suggested promise for a technique combining the use of large amounts of occurrence data with small amounts of abundance data, but none have yet performed well enough to be of use for many real‐world applications.

We present a two‐stage modeling approach for predicting abundance, where the results of SDMs produced using the R package biomod2 (Thuiller, Georges, Engler, & Breiner, 2016) are regressed against abundance data and additional predictors using random forest regression with the R packages caret and randomForest (Breiman, 2001; Kuhn et al., 2016; Liaw & Wiener, 2002). This approach performs well in almost all cases tested here and is flexible and simple to use. We argue that poor correlations between SDM results and abundance previously reported may be partly due to the use of less powerful or inappropriate modeling techniques in other studies. SDMs are first produced using, in our case, presence–absence data to produce a map of estimated probability of occupancy for the species of interest. Separate abundance data are then used to fit a random forest regression that predicts abundance from probability of occupancy. Additional predictors, which may be expected to influence abundance but not occupancy, can be included at this stage. We also include the SDM results of co‐occurring species as covariates in the random forest regression, allowing biotic effects to be accounted for in the prediction of abundance and producing more realistic species responses.

We have used this approach to produce distribution maps showing the abundance of 20 common tree species in Great Britain. Available tree distribution data for Great Britain were surprisingly poor, presenting a knowledge gap for ecologists working on British woodlands, particularly in light of major threats such as emerging tree diseases (Boyd, Freer‐Smith, Gilligan, & Godfray, 2013). Our distributions show total combined area covered by each species within each square kilometer (hectares per square kilometer) across Great Britain and are a significant improvement on previously widely available distribution data. We envisage that such distribution maps could make an important contribution in a number of fields related to British forestry, from conservation planning to epidemiology.

## 2 Methods

We predicted abundance of tree species using a combination of two established techniques. First, we used the R package biomod2 (Thuiller et al., 2016) to produce ensemble species distribution models (SDMs) of trees in Great Britain at 1 km^2^ resolution. Then, we used random forest regression, with caret (Kuhn et al., 2016) and randomForest (Liaw & Wiener, 2002) packages in R, to link the results of these SDMs to a much smaller amount of abundance data, to predict abundance across Great Britain at the same resolution.

### 2.1 Stage 1: Fitting species distribution models

From the Distribution Database of the Botanical Society of the British Isles (BSBI) (see Data Accessibility), we downloaded all records from Great Britain between 1950 and 2014 for 25 commonly found tree species: *Acer campestre* L., *Acer platanoides* L., *Acer pseudoplatanus* L., *Alnus glutinosa* (L.) Gaertner, *Betula pendula* Roth, *Betula pubescens* Ehrhart, *Carpinus betulus* L., *Castanea sativa* Miller, *Corylus avellana* L., *Crataegus monogyna* von Jacquin, *Fagus sylvatica* L., *Fraxinus excelsior* L., *Populus tremula* L., *Prunus avium* L., *Prunus padus* L., *Pseudotsuga menziesii* Franco, *Quercus petraea* Lieblein, *Quercus robur* L., *Salix caprea* L., *Salix cinerea* L., *Sorbus aria* Crantz, *Taxus baccata* L., *Tilia cordata* Miller, *Ulmus glabra* Hudson, and *Ulmus procera* Salisbury. We discarded records with location data less precise than tetrad level (2 × 2 km) and simplified data with more precise locations to tetrad level. We chose tetrad resolution as a suitable compromise between having a high number of records to use and a small spatial scale, as using coarse scales can be problematic when modeling species distributions (Dengler, Löbel, & Dolnik, 2009; Guisan, Graham et al., 2007).

We then converted this presence‐only data to presence–absence. We considered tetrads for which botanical surveys had been undertaken at least twice since 1950, and where at least 50 species of plants were recorded in each survey, to be “well‐surveyed” (Groom, 2013) (a map is provided in Appendix 1). Any well‐surveyed tetrads that did not have records for the species of interest were reclassified as “absence” points for that species (i.e., locations where the species was likely to be either truly absent or at very low abundance and therefore playing little role in defining the dominant ecological characteristics of that tetrad). Accounting for the likelihood that common trees will have a higher detection probability than most species of plants, we kept this threshold low enough to prevent the exclusion of tetrads in species‐poor areas, while being high enough to prevent the inclusion of too many poorly surveyed tetrads (Groom, 2013). This produced a total of 18,993 tetrads from across Great Britain that were considered well surveyed and subsequently used as presence or absence points. Data manipulation was carried out using custom‐written scripts in Python (Python Software Foundation: Version 3.3.2).

We downloaded data on a variety of ecological variables across Great Britain from a variety of free sources (Table 1). See Data Accessibility for details. Preprocessing of layers was carried out in ArcGIS (ESRI 2014) to ensure identical extent, cell size, and coordinate system for use in species distribution modeling. All environmental covariates were used at 1 km resolution: vector datasets were rasterized to 1 km resolution.

**Table 1:** Ecological variables downloaded and produced for species distribution modeling. Details of data sources can be found in Data Accessibility

Variable	Description	Unit	Source
bio1	Annual mean temperature	°C × 10	Worldclim
bio2	Mean diurnal temperature range: mean of monthly (max temp − min temp)	°C × 10	Worldclim
bio3	Isothermality (bio2/bio7 × 100)	°C × 10	Worldclim
bio4	Temperature seasonality: standard deviation × 100	°C × 1000	Worldclim
bio5	Max temperature of warmest month	°C × 10	Worldclim
bio6	Min temperature of warmest month	°C × 10	Worldclim
bio7	Temperature annual range	°C × 10	Worldclim
bio8	Mean temperature of wettest quarter	°C × 10	Worldclim
bio9	Mean temperature of driest quarter	°C × 10	Worldclim
bio10	Mean temperature of warmest quarter	°C × 10	Worldclim
bio11	Mean temperature of coldest quarter	°C × 10	Worldclim
bio12	Annual precipitation	mm	Worldclim
bio13	Precipitation of wettest month	mm	Worldclim
bio14	Precipitation of Driest Month	mm	Worldclim
bio15	Precipitation seasonality: coefficient of variation	cm	Worldclim
bio16	Precipitation of wettest quarter	mm	Worldclim
bio17	Precipitation of driest quarter	mm	Worldclim
bio18	Precipitation of warmest quarter	mm	Worldclim
bio19	Precipitation of coldest quarter	mm	Worldclim
altitude	Altitude	m × 10	Worldclim
slope	Slope	%	Derived from Altitude using ArcGIS (Slope)
aspect	Aspect	Degrees	Derived from Altitude using ArcGIS (Slope)
directradiat	Direct radiation: incoming direct solar radiation	Watt hr m^−2^	Derived from Altitude using ArcGIS (Solar Radiation Analysis)
directdurat	Direct duration: duration of direct solar radiation	Hours	Derived from Altitude using ArcGIS (Solar Radiation Analysis)
diffuseradiat	Diffuse radiation: incoming scattered solar radiation	Watt hr m^−2^	Derived from Altitude using ArcGIS (Solar Radiation Analysis)
nfi	National Forest Inventory Great Britain 2014, forested areas	Nominal	Forestry Commission
soil	Soil type	Nominal	European Soil Database
soiltext	Dominant soil surface textural class	Nominal	European Soil Database
octop	Topsoil organic carbon content	Nominal	European Soil Database
awctop	Topsoil available water capacity	Nominal	European Soil Database
mintop	Topsoil minerology	Nominal	European Soil Database
ancient_es	Ancient woodlands in England, Scotland and Wales	Nominal	Natural England, Forestry Commission Scotland and National Resources Wales
land cover 07	UK Land cover map 2007 (1 km^2^)	Nominal	Countryside Survey/CEH

We then fitted species distribution models (SDMs) to these data. For reviews of these methods, see Elith and Leathwick (2009) and Pearson and Dawson (2003). SDMs use species records and environmental variables to fit models that describe the relationship of the species’ distribution to the environmental variables, which can then be used to predict the occupancy probability or related measures across a wider landscape (Elith & Leathwick, 2009; Thuiller, 2003). SDMs for all species were produced using the package biomod2 in R (R Core Team 2015; Thuiller et al., 2016).

We selected 15 environmental variables as covariates from the original set of 33. We removed one of each pair of variables with a pairwise Pearson's correlation coefficient higher than 0.7, while retaining variables that are known to be important determinants of plant growth (Guisan, Zimmermann et al., 2007; Prentice et al., 1992). The final selection was altitude, aspect, slope, direct incoming solar radiation, mean diurnal temperature range, temperature seasonality, annual precipitation, ancient woodland locations, topsoil available water capacity, topsoil minerology, topsoil organic carbon content, topsoil texture class, soil category, National Forest Inventory (NFI) forest type, and land cover type(see Appendix 2 for pairwise Pearson's correlations between selected variables). We ran six algorithms (GLM, GAM, classification tree analysis (CTA), generalized boosting models (GBM), random forest (RF), and maximum entropy (MaxEnt)) 15 times for each species using the 15 environmental covariates, producing a total of (25 species × 6 algorithms × 15 repeats) = 2,250 models.

Each model run was carried out using a randomly chosen 70% of the presence–absence data (Heikkinen, Marmion, & Luoto, 2012; Thuiller, 2003); the remaining 30% were used for cross‐validation to assess the performance of each model using two model assessment criteria; area under the receiver operator curve (ROC) and the true skill statistic (TSS; Allouche, Tsoar, & Kadmon, 2006). For each species, we selected the best‐performing models (see Table 2) to build an ensemble distribution model (a mean of the raw model results, weighted by the model ROC scores), producing a single distribution map for each species that represents a robust estimate of a species’ British distribution at 1 km^2^ resolution (Thuiller et al., 2016). The model selection process was as follows. Firstly, we assessed ROC and TSS scores—for both metrics, a higher value indicates better model fit—and if there was a leading group of models whose ROC and TSS scores were a step higher than the remainder, this leading group was chosen. Often this leading group contained just the 15 random forest models. Otherwise, the top 20 models with the highest scores were selected. Secondly, we visually assessed the predicted responses of the species to each environmental covariate for each of these models. Any models that contained biologically implausible responses were rejected, as were models where the responses or predicted occurrence maps disagreed greatly from the overall consensus, as these can lead to development of inappropriate ensemble models (H. Hannemann, personal communication). See the walkthrough of R code in Supporting Information for an example of how models were chosen and example response curves. After rejection of implausible models, the final number of models used to produce each ensemble ranged between 11 and 20. Ensemble models were therefore robust, biologically plausible, and had high predictive power for the majority of species (see Table 2).

**Table 2:** The number, type, and prediction accuracy of the individual models used to build ensemble distribution models for each tree species. Algorithms included were GAM (generalized additive model), GBM (generalized boosted regression), GLM (General Linear Model), RF (Random Forest), and MaxEnt (Maximum Entropy)

Species	Number of models used to build ensemble	Algorithms included	Mean ROC score	Mean TSS score
*Acer campestre*	20	GAM, RF, GBM	0.92	0.71
*Acer platanoides*	20	GLM, GAM, RF, GBM	0.76	0.44
*Acer pseudoplatanus*	20	GAM, RF, GBM	0.85	0.55
*Alnus glutinosa*	15	RF	0.80	0.46
*Betula pendula*	15	RF	0.79	0.46
*Betula pubescens*	15	RF	0.80	0.46
*Carpinus betulus*	20	RF, GBM, MaxEnt	0.78	0.40
*Castanea sativa*	15	RF	0.81	0.47
*Corylus avellana*	16	RF, GBM	0.86	0.46
*Crataegus monogyna*	20	GLM, GBM, RF, GBM	0.96	0.82
*Fagus sylvatica*	20	GAM, RF, GBM	0.81	0.48
*Fraxinus excelsior*	20	GLM, GAM, RF, GBM	0.92	0.83
*Populus tremula*	17	RF, GBM	0.71	0.31
*Prunus avium*	11	RF	0.75	0.36
*Prunus padus*	20	RF, GBM	0.80	0.48
*Pseudotsuga menziesii*	19	GAM, RF, GBM, MaxEnt	0.76	0.39
*Quercus petraea*	15	RF	0.82	0.49
*Quercus robur*	16	RF, GBM	0.90	0.64
*Salix caprea*	16	RF, GBM	0.79	0.42
*Salix cinerea*	16	RF, GBM	0.78	0.42
*Sorbus aria*	20	RF, GBM	0.84	0.53
*Taxus baccata*	20	GAM, RF, GBM	0.80	0.44
*Tilia cordata*	15	RF, GBM	0.76	0.36
*Ulmus glabra*	15	RF	0.79	0.43
*Ulmus procera*	15	RF	0.89	0.61

Nonsignificant variables were not removed from the models because of the very large size of our datasets, and because the models were used to make predictions rather than to test hypotheses. Therefore, final models may include terms that were not important to the outcome, but this should not have had a detrimental impact on the model fit. The numbers and types of models selected for each species are displayed in Table 2.

### 2.2 Stage 2: Modeling abundance using random forest regression

Abundance data for trees, in the form of hectares covered by a species per square kilometer (or percent cover), were obtained from the Countryside Survey and myForest (see Data Accessibility). The Countryside Survey is a large‐scale survey in Great Britain measuring many aspects of landscapes and the countryside, including diversity and abundance of plant species. It uses a random stratified sampling procedure to capture a representative sample of all land cover types. By contrast, myForest is a service set up to help woodland owners map and manage their forests, which currently holds data on over 45,000 ha of woodlands across Great Britain, but does not contain any records outside of woodlands. For all tree species combined, 9,800 randomly selected abundance data points from the Countryside Survey and 9,453 abundance data points from myForest were used, making an average of 770 abundance data points per species (see Appendix 5 for numbers of data points per species).

The two abundance datasets (Countryside Survey and myForest) were rescaled to express them as hectares covered per kilometer squared (percent cover), in order to make them comparable. For the myForest data, which was originally provided in the format percentage cover of each species within a woodland patch, this involved multiplying each percentage cover record by the proportion of woodland cover in the relevant kilometer square. For this, we used a shapefile downloaded from the National Forest Inventory (NFI), containing outlines of all woodlands over 0.5 ha in Great Britain. For the Countryside Survey data, which was collected using a more complex methodology (details available in Barr et al., 1993) where linear features such as hedgerows were sampled separately from the rest of the landscape, more manipulation was required. The data were weighted by the length of linear features in the kilometer squared, to account for the fact that linear features are more likely to contain trees and the lengths of them are not equal across the country. The weighting was done using (linear plot percentage cover × percent of kilometer square covered by linear features) + (nonlinear plot percentage cover × remaining area), with all required information taken from the Countryside Survey.

Tree cover data for England and Wales from Bluesky's National Canopy Map were made available by the Woodland Trust, to be used as a modeling covariate. Three layers from this were used: the total tree cover, tree cover derived only from woodlands included in the NFI, and tree cover derived from trees outside woodlands. The National Canopy Map layers were used in England and Wales, while the more basic NFI layers were used in Scotland where complete tree cover data were not available. We also used the NFI dataset to calculate the proportion of each square taken up by broadleaved woodland edge, which was defined as any woodland within 50 m of nonwoodland (Aune, Gunnar, & Moen, 2005). All these layers were used as covariates in the random forest regression (below). We used R version 3.2.3 for all modeling and data processing (R Core Team 2015).

We used random forest regression to model the relationships between abundance, the probability of occupancy predicted by the SDMs, and our tree cover covariates which we expected would be important for modeling tree abundance (Breiman, 2001). A separate random forest regression was implemented for each species. The SDM outputs for all species were included as variables for each species, so that the models would also capture interactions between species (such as competition). Potentially, this could also capture variation in other variables that are not included in that species’ SDM but which correlate with the distribution of other species. Models had the form:

$${\text{Abundance}}_{\mathrm{focal}\mathrm{sp}.}∼{\hat{P}}_{\mathrm{focal}\mathrm{sp}.}+{\hat{P}}_{\mathrm{sp}.2}\ldots {\hat{P}}_{\mathrm{sp}.25}+C_{A}+C_{W}+C_{O}+C_{E}$$

where *P̂* is the predicted probability of occupancy from the relevant SDM, C_A_ is cover from all trees, C_W_ is cover from woodland trees only, C_O_ is cover from trees outside woodland only, and C_E_ is cover from woodland edge.

**Figure 1:**
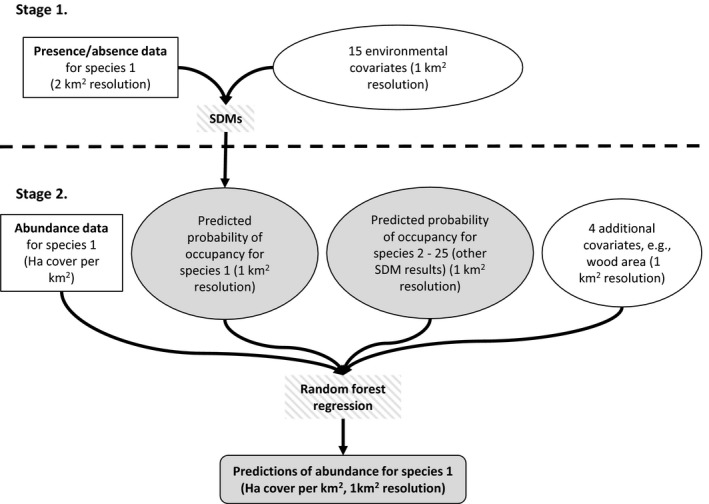
Schematic showing the outline of the two‐stage method for predicting abundance distributions. The first stage uses SDMs to produce maps of predicted probability of occupancy, while the second stage takes these maps as inputs and uses Random Forest regression to produce maps of predicted abundance. Distribution data inputs are shown in square boxes and model covariates in round boxes, and model outputs are shaded in solid gray and modeling processes in hashed gray

Models were run using the combined myForest and Countryside Survey data. We chose to use random forest regression because it is insensitive to data distribution and therefore copes well with our data which has a high percentage of zeros. It can also take a large number of potentially collinear variables, and is robust to overfitting, making it extremely useful for prediction (Prasad, Iverson, & Liaw, 2006; Segal, 2004). We used these models to predict abundance of each species across the whole of Great Britain at 1 km^2^ resolution. We used root‐mean‐square error (RMSE) and mean absolute error (MAE), produced by *k*‐fold cross‐validation with 10‐fold, to evaluate our models. These two commonly used evaluation metrics give interpretations of a model's average error when testing it against independent data, in this case, the 10% that was left out of each run (Chai & Draxler, 2014). A schematic overview of the whole two‐stage method is shown in Figure 1.

## 3 Results

### 3.1 Species distribution modeling

All selected models had useful prediction capability (AUC > 0.7) (Boyce, Vernier, Nielsen, & Schmiegelow, 2002). In general, prediction accuracy of the selected models was good and they successfully predicted a large proportion of known presence or absence points. The selected models had ROC scores between 0.71 and 0.96 and TSS scores between 0.31 and 0.83 (Table 2). Ensemble models were built using 100% of the available data, so evaluations are not given for ensemble models as this would test the models on the same data they were generated with, resulting in unfair evaluation statistics. For the four species with the lowest predictive power (lowest TSS and ROC scores), (*Populus tremula*,* Prunus avium*,* Pseudotsuga menziesii,* and *Tilia cordata*) (Table 2), we investigated further to ensure that all ecological factors known to be important to them were included in the model runs. However, no further improvements to the model fit were found. These were species that tend to be either widespread but uncommon throughout their range (*P. tremula*,* P. avium*,* T. cordata*) or non‐native trees whose distribution is largely controlled by human planting (*P. menziesii*), and as a result, it is unlikely to be possible to generate high‐scoring distribution models for these species (Guisan, Zimmermann et al., 2007). For 21 of 25 species, however, SDMs produced high‐quality ensemble models.

### 3.2 Abundance modeling

**Figure 2:**
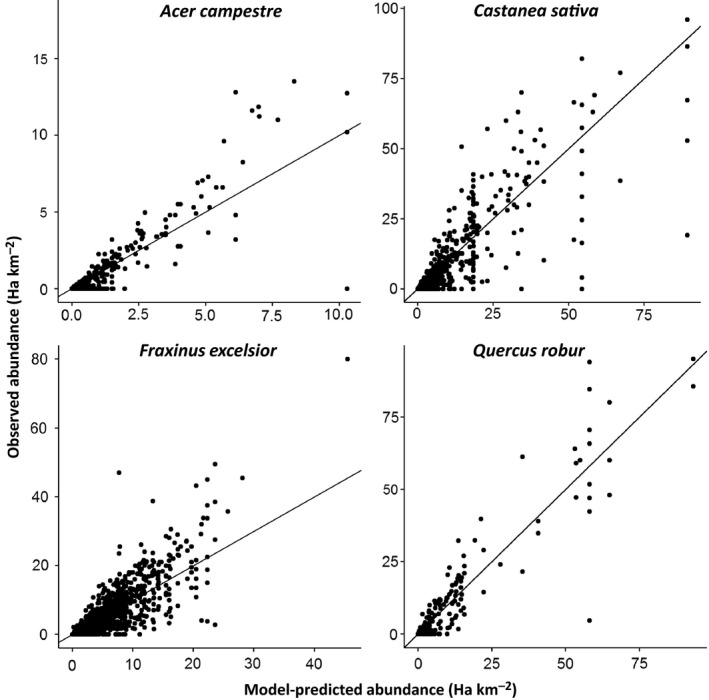
Observed abundance against abundance predicted by Random Forest regression, as used to assess model performance, shown for four tree species. The line on each graph is the 1:1 line showing perfect model fit

In general, the random forest models were very successful in predicting the abundance of tree species. Figure 2 shows the predicted against observed abundance for four representative species; graphs for all other species are included in Appendix 3. For the majority of species, the predictions of the models are similar to the observed values.

We produced root‐mean‐square error (RMSE) and mean absolute error (MAE) scores using 10‐fold cross‐validation to evaluate our models’ performance. These two commonly used model evaluation metrics give interpretations of a model's average error when testing it against independent data (Chai & Draxler, 2014). Table 3 shows RMSE and MAE scores for each species; the error scores are given in the same scale as the response variable, that is, hectares covered per square kilometer (percent cover). All the models have RMSE scores under 10, and most are under 5. All MAE scores are under 5. The average prediction error for most of the models produced is therefore <5%.

**Table 3:** Root‐mean‐square error (RMSE) and mean absolute error (MAE) scores for the Random Forest regression model for each species. The number of nonzero data points available for each species is also shown

Species	RMSE	MAE	Number of nonzero data points
*Acer campestre*	1.44	0.35	315
*Acer platanoides*	1.27	0.19	42
*Acer pseudoplatanus*	4.01	1.40	634
*Alnus glutinosa*	2.40	0.66	195
*Betula pendula*	6.88	2.29	802
*Betula pubescens*	4.09	1.09	127
*Carpinus betulus*	3.79	1.05	320
*Castanea sativa*	9.56	3.58	501
*Corylus avellana*	4.47	1.47	935
*Crataegus monogyna*	1.10	0.23	339
*Fagus sylvatica*	8.45	2.91	918
*Fraxinus excelsior*	4.95	1.88	1629
*Populus tremula*	*NA*	*NA*	16
*Prunus avium*	1.98	0.56	401
*Prunus padus*	*NA*	*NA*	9
*Pseudotsuga menziesii*	7.66	1.96	193
*Quercus petraea*	5.99	1.84	209
*Quercus robur*	6.50	2.54	1867
*Salix caprea*	1.38	0.28	74
*Salix cinerea*	0.16	0.03	55
*Sorbus aria*	*NA*	*NA*	3
*Taxus baccata*	2.21	0.49	86
*Tilia cordata*	1.04	0.14	56
*Ulmus glabra*	*NA*	*NA*	22
*Ulmus procera*	*NA*	*NA*	27

**Figure 3:**
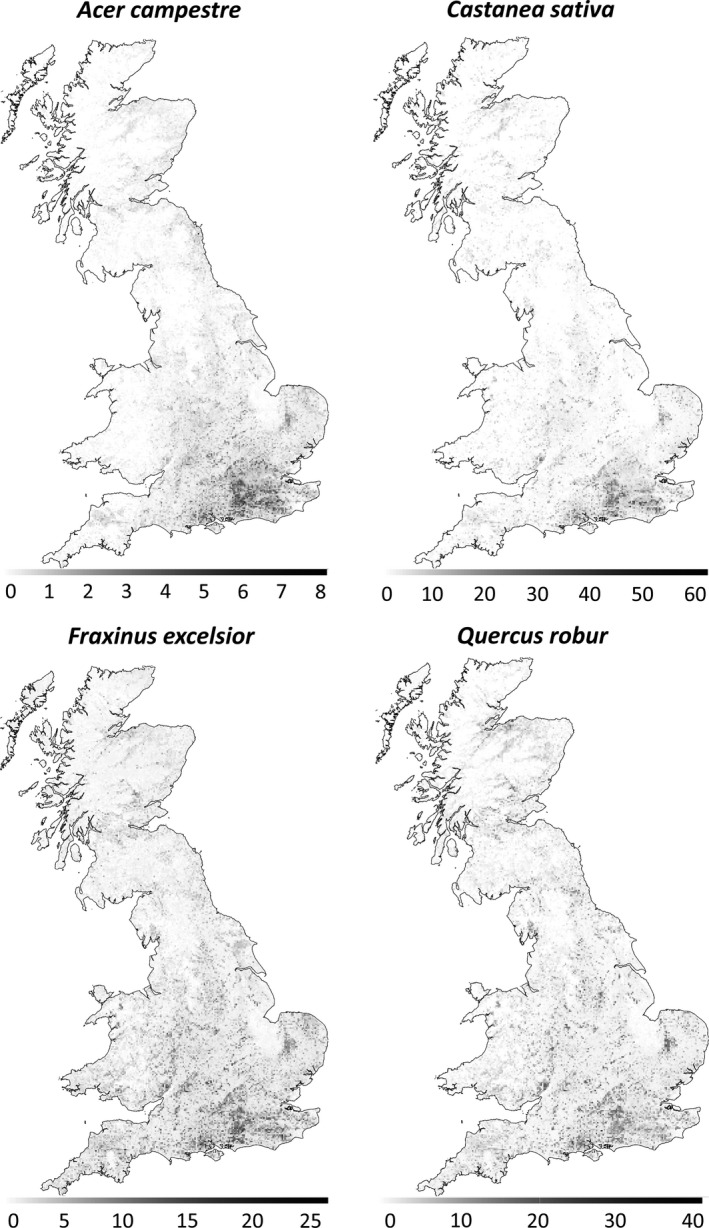
Maps of predicted abundance for four species, in hectares per km^2^, or percent cover. Note the scale varies between species. Maps for all other successfully modeled species are available to download from Sylva Foundation website and Oxford University Research Archive (see Data Accessibility)

For six species, *Acer platanoides*,* Populus tremula*,* Prunus padus*,* Sorbus aria*,* Ulmus glabra,* and *Ulmus procera*, there were too few nonzero abundance data points to use 10‐fold cross‐validation. We chose 50 positive data points as the cutoff for using 10‐fold cross‐validation, as this gives an average of five nonzero data points per fold. *Acer platanoides* had 42 positive abundance data points, so for this species, we used eightfold cross‐validation to maintain an average of five nonzero data points per fold. However, for the remaining five species, we felt that there was not enough data available to produce reliable abundance models (see Table 3). These species were omitted, and maps of predicted abundance of the remaining 20 species across Great Britain were produced (Figure 3 and downloadable from the Sylva Foundation website and Oxford University Research Archive (see Data Accessibility). Where adequate abundance data were available, however, random forest regression was able to improve the prediction of the species for which the SDMs had a poorer fit. We were able to successfully model the abundance of *Prunus avium*,* Pseudotsuga menziesii,* and *Tilia cordata* despite the SDM prediction accuracy for these species being poorer than the other species.

We also calculated *R* ^2^ scores for the models, and these are available in Appendix 5. However, we recommend caution when interpreting these scores, as *R* ^2^ is not the most appropriate metric to use in this situation. *R* ^2^ is affected by the extent of the dependent variable (Gelman & Hill, 2007), and as the maximum abundance varied greatly between species, this confounds comparison between our models. The high percentage of zeros in our datasets also produces difficulty in the interpretation of *R* ^2^. For instance, for *Acer campestre*, over 97% of the available abundance data points were zero. The model tended to predict very slightly higher than zero for these points (generally between zero and one percent cover), resulting in a low *R* ^2^ (.523). However, the observed vs predicted graph (Figure 2) and the low RMSE and MAE scores (Table 3) for *Acer campestre* show that the model generally predicts very close to the true abundance, despite scatter in the data, and this is mirrored for most other species. For applications where the difference between zero and one or two percent cover is unimportant, these models can be used directly for predicting abundance; where it is more important, the predicted against observed graph can be used to select a cutoff below which predicted abundance will be coerced to zero.

## 4 Discussion

The two‐stage modeling approach produced good or excellent predictions of abundance for the majority of species across the whole of Great Britain, despite only being trained on a relatively small amount of abundance data. This is in contrast to several previous studies looking for relationships between SDM results and abundance, which have shown little or no relationship (Gaston et al., 2000; Johnson & Seip, 2008; Nielsen, Johnson, Heard, & Boyce, 2005). However, to our knowledge, no previous studies have used random forest regression to model this relationship, and doing so has a number of advantages. Most importantly perhaps is that it does not make any assumptions about the shape of the relationship. Previous studies have attempted to use the negative binomial and other theoretical distributions, but we argue that this is likely to be an oversimplification that may mask true relationships. The shape of such a relationship, which is likely to have several different drivers, may not follow a simple mathematical function, and is known to vary between species (Gaston et al., 2000; Harris, 2015; Nielsen et al. 2005). The use of random forest regression allows for such variation, making it a much more powerful technique for this application (De'ath & Fabricius, 2000; Evans & Cushman, 2009; Prasad et al., 2006).

Our two‐stage modeling approach has a number of other advantages. It can incorporate biotic effects, and include covariates that are expected to influence abundance separately from those expected to influence occupancy. It makes use of the large amount of presence or presence–absence data that are often available, rather than discarding it. It will work with any measure of abundance (number of individuals, percentage cover, biomass, *etc*.) and has been shown to be effective over large spatial extents. It may be a particularly powerful approach where occurrence and abundance are not influenced by exactly the same factors (see Nielsen et al., 2005). Although not tested here, this method also has the potential to be effective when used with the results of more problematic SDMs, such as those made using presence‐only data, which can only predict a relative likelihood of occupancy (Araújo & Peterson, 2012).

We can also make use of the covariate allocation of random forest to gain insights into underlying ecological processes within the community. For each species for which we have modeled abundance, we can inspect which variables are having the strongest effects in the model (see Appendix 4) (Breiman, 2001). This means we can see which other species’ SDM results are most strongly associated with the abundance of our species of interest, allowing us to identify possible biotic interactions such as competition. This does not allow us to distinguish causal relationships because of the possibility that hidden covariates could be at play; two species’ SDM results could be correlated with each other not because of a biotic interaction, but because they are both influenced by an underlying factor. However, it does provide a qualitative estimate of biotic effects that could be an interesting starting point for further study. The inclusion of biotic effects may have the additional benefit of improving model performance for predicting abundance under new conditions, such as future climate scenarios (Anderson, 2013; Araújo & Guisan, 2006; Elith & Leathwick, 2009; Harris, 2015). Species distributions and abundances are predicted to be strongly influenced in future by both climatic changes and biotic effects, and to our knowledge, this is the first technique for predicting abundance which is able to make some account of these biotic effects. However, the approach will not be able to incorporate changes to biotic effects with novel species assemblages, or other factors such as dispersal limitation, without further modification.

Not all species were successfully modeled using this technique. *Prunus padus*,* Populus tremula*,* Sorbus aria*,* Ulmus glabra,* and *Ulmus procera* were all unsuccessful, in each case because very little abundance data were available for these species in our datasets. For example, our combined abundance dataset contained only four nonzero data points for *Sorbus aria*, demonstrating the difficulty in acquiring abundance data even for such a well‐studied system. However, various tree species which are generally considered to be difficult to model—such as *Pseudotsuga menziesii*, a non‐native species whose distribution is still largely controlled by planting, and *Tilia cordata*, which is thought to be both rare and widespread in Britain due to an unusual ecological history (Pigott, 1991)—were successfully modeled by random forest regression, despite showing relatively poor SDM performance. Overall, the method performed well for the majority of species and seems to be generally effective across a range of species, provided that sufficient abundance data are available.

British trees exist in highly human‐modified landscapes where their distributions have without exception been altered by human land use and preferences (Hopkins & Kirby, 2007; Rackham, 2008). This is a challenging scenario for modeling abundance; other studies which have tried to model abundance of vascular plant species have avoided trees for this reason (Van Couwenberghe et al., 2013). However, despite this, the models performed well for the majority of species. This suggests that the models may be flexible enough to work in a variety of contexts and are likely to perform even better in less human‐dominated landscapes. This flexibility is one of the major advantages of using random forest regression, and we expect it to offer broad application in modeling abundance of a wide range of species (Prasad et al., 2006). The next step for evaluating the method will be to compare its performance to other published methods for predicting abundance, which could be done by evaluating the relative performance of this and other methods with a variety of published datasets.

**Figure 4:**
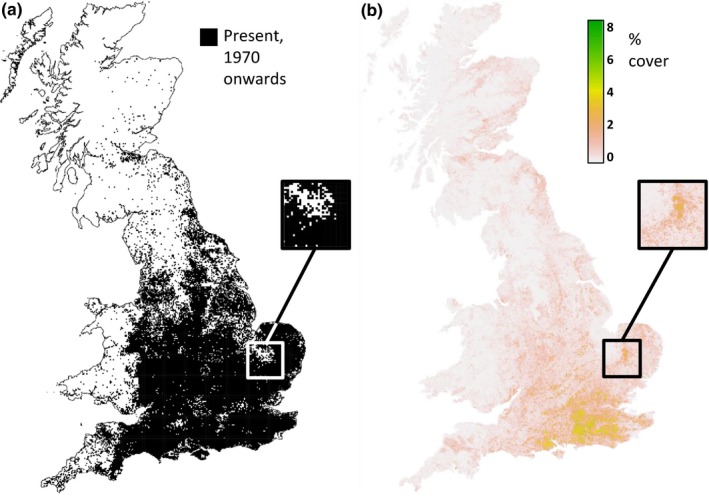
(a) Presence records of *Acer campestre*, downloaded from the BSBI database (some of the best available distribution data at the country‐wide level). The data are presence only on 2 × 2 km (tetrad) scale. Note that where presence is not recorded, it is impossible to say whether the species is truly absent. Compare with our modeled abundance distribution (b) showing modeled hectares covered by *A. campestre* per square kilometer for every 1 km square

The abundance maps that we have produced are the best quality abundance distributions currently available for these species in Great Britain; previously, the best widely available distribution maps for trees in Great Britain were presence‐only maps on 10‐ or 2‐km square scales (see Figure 4). Our maps are modeled, not directly observed, and as is the case for modeling any highly noisy system, will not accurately predict abundance in every 1 km square; however, they are expected to capture overall patterns of distribution well. As more data, particularly abundance data and better quality environmental covariates, become available, our maps can continue to be improved. Abundance maps have a far wider variety of applications than presence‐only maps, and these maps will allow significant improvements to these applications. British woods face a range of threats, including invasive diseases such as ash dieback, undermanagement or overmanagement leading to poor woodland quality, pollution, and damage by deer (Rackham, 2008). These improved maps should allow better planning and management of woodlands, analysis of habitats and ecosystem functioning, and epidemiology and disease management, and will be a useful contribution to the protection of British trees.

## 5 Conclusion

The two‐stage method to predict abundance, using random forest regression to model the relationship between SDM outputs and abundance, is robust and easy to use producing good results for the majority of British tree species. Images and raster files of our abundance maps for the 20 successfully modeled tree species are available to download from the Sylva Foundation website and Oxford University Research Archive (see Data Accessibility). Both SDMs and random forest regression are well‐established techniques, and using them together in this combination is a user‐friendly way to produce good‐quality maps of predicted abundance. This opens the way for more abundance maps to be produced for a wider range of scenarios, which itself could drive improvements in a number of ecological research areas, from responses to climate change to epidemiology. To facilitate this, we provide annotated R code in the Supporting Information for the entire process, to act as a guide for those wishing to use this method themselves.

## Data Accessibility

- Ancient Woodland shapefile data:
  ○ England: available from Natural England at http://www.gis.naturalengland.org.uk/pubs/gis/GIS_register.asp (accessed 17/06/2016).
  ○ Scotland: available from Forestry Commission Scotland at https://gateway.snh.gov.uk/natural-spaces/dataset.jsp?dsid=AWI (accessed 17/06/2016).
  ○ Wales: available from National Resources Wales at http://lle.wales.gov.uk/Catalogue/Item/AncientWoodlandInventory2011/?lang=en (accessed 17/06/2016).
- BSBI Distribution Database: available at http://bsbidb.org.uk/ (accessed 17/06/2016).
- Countryside Survey data: the data used is available to download from the Oxford University Research Archive, https://ora.ox.ac.uk/ “Merged abundance dataset from myForest and the Countryside Survey”.
- Landcover Map 2007: available from Centre for Ecology and Hydrology at http://www.ceh.ac.uk/services/land-cover-map-2007 (accessed 17/06/2016).
- myForest: the data used is available to download from the Oxford University Research Archive, https://ora.ox.ac.uk/ “Merged abundance dataset from myForest and the Countryside Survey”.
- National Canopy Map was made available for this study by kind permission of the Woodland Trust.
- National Forest Inventory Great Britain 2014: available from Forestry Commission at http://www.forestry.gov.uk/datadownload (accessed 17/06/2016).
- R scripts: see Supporting Information.
- Raster and image files for the abundance maps produced for all species are available to download from the Sylva Foundation website, https://sylva.org.uk/ and Oxford University Research Archive, https://ora.ox.ac.uk/ “Predicted abundance maps for British Trees”.
- Soil data from European Soil Database: available at http://esdac.jrc.ec.europa.eu/content/european-soil-database-v20-vector-and-attribute-data (accessed 17/06/2016).
- Worldclim: free global climate data, available at http://www.worldclim.org/ (accessed 17/06/2016).

## Conflict of Interest

None declared.

## Appendix 1

### 1.1

Map of well‐surveyed tetrads (black) that were used to convert presence‐only data into presence–absence data. A total of 18,993 tetrads were found to have been surveyed at least twice since 1950, with at least 50 species of plants recorded in each survey. These tetrads were considered to be well surveyed enough that if a tree species had not been recorded in one, it was considered very likely to be either truly absent or present at very low abundance with low ecological importance in the tetrad, and was therefore classified as an “absence”

## Appendix 2

### Pearson's pairwise correlations for the numeric variables used in species distribution modeling. The cutoff for correlation coefficient used for variable selection was 0.7

**Table:**

	Altitude	Aspect	Slope	Direct incoming solar radiation	Mean diurnal temperature range	Temperature seasonality	Annual precipitation	Topsoil available water capacity	Topsoil minerology	Topsoil organic carbon content	Topsoil texture class
Altitude	1	0.015396	0.518652	0.591347	−0.20693	−0.03162	0.621356	0.231888	0.000805	0.303198	0.428471
Aspect	0.015396	1	−0.02016	0.062394	−0.05345	−0.0269	0.062306	0.014767	0.019412	0.035133	0.041259
Slope	0.518652	−0.02016	1	0.224245	−0.08178	0.00709	0.331202	0.104236	−0.01246	0.154548	0.180757
Direct incoming solar radiation	0.591347	0.062394	0.224245	1	−0.30348	−0.17634	0.627365	0.225541	0.055276	0.231702	0.407135
Mean diurnal temperature range	−0.20693	−0.05345	−0.08178	−0.30348	1	0.620974	−0.57396	−0.0862	0.068989	−0.26488	−0.27498
Temperature seasonality	−0.03162	−0.0269	0.00709	−0.17634	0.620974	1	−0.37328	−0.13755	0.054918	−0.21352	−0.27218
Annual precipitation	0.621356	0.062306	0.331202	0.627365	−0.57396	−0.37328	1	0.27932	0.039224	0.36968	0.506227
Topsoil available water capacity	0.231888	0.014767	0.104236	0.225541	−0.0862	−0.13755	0.27932	1	0.152653	0.305173	0.590749
Topsoil minerology	0.000805	0.019412	−0.01246	0.055276	0.068989	0.054918	0.039224	0.152653	1	0.142262	0.298536
Topsoil organic carbon content	0.303198	0.035133	0.154548	0.231702	−0.26488	−0.21352	0.36968	0.305173	0.142262	1	0.386809
Topsoil texture class	0.428471	0.041259	0.180757	0.407135	−0.27498	−0.27218	0.506227	0.590749	0.298536	0.386809	1

## Appendix 3

### 3.1

Observed abundance against abundance predicted by Random Forest regression, as used to assess model performance. The line on each graph is the 1:1 line showing perfect model fit. From top left, species are *Acer campestre*,* Acer platanoides*,* Acer pseudoplatanus*,* Alnus glutinosa*,* Betula pendula*,* Betula pubescens*,* Carpinus betulus*,* Castanea sativa*,* Corylus avellana*,* Crataegus monogyna*,* Fagus sylvatica*,* Fraxinus excelsior*,* Populus tremula*,* Prunus avium*,* Prunus padus*,* Pseudotsuga menziesii*,* Quercus petraea*,* Quercus robur*,* Sorbus aria*,* Salix caprea*,* Salix cinerea*,* Taxus baccata*,* Tilia cordata*,* Ulmus glabra,* and *Ulmus procera*

## Appendix 4

### The most important variables in the random forest regressions of abundance for each species. Full variance importance plots for each species are available from the authors on request

**Table:**

Species	Most important variable in abundance model
*Acer campestre*	Cover of trees in NFI
*Acer platanoides*	Probability of occupancy of *Crataegus monogyna*
*Acer pseudoplatanus*	Cover of trees in NFI
*Alnus glutinosa*	Probability of occupancy of *Crataegus monogyna*
*Betula pendula*	Cover of all trees
*Betula pubescens*	Cover of trees outside of NFI
*Carpinus betulus*	Cover of all trees
*Castanea sativa*	Cover of trees in NFI
*Corylus avellana*	Cover of trees in NFI
*Crataegus monogyna*	Probability of occupancy of *Betula pendula*
*Fagus sylvatica*	Cover of all trees
*Fraxinus excelsior*	Cover of trees in NFI
*Prunus avium*	Probability of occupancy of *Prunus avium*
*Pseudotsuga menziesii*	Cover of trees in NFI
*Quercus petraea*	Cover of trees outside of NFI
*Quercus robur*	Cover of trees in NFI
*Salix caprea*	Probability of occupancy of *Fagus sylvatica*
*Salix cinerea*	Probability of occupancy of *Corylus avellana*
*Taxus baccata*	Cover of trees in NFI
*Tilia cordata*	Probability of occupancy of *Fagus sylvatica*

## Appendix 5

### Additional information about abundance models. *R* ^2^ scores are shown for each species, along with RMSE (root‐mean‐square error) and MAE (mean absolute error) scores. For information about interpreting *R* ^2^ scores, see main text. Number of abundance data points per species and number of nonzero abundance data points per species are also shown

**Table:**

Species	*R* ^2^	RMSE	MAE	Number of data points per species	Number of nonzero data points
*Acer campestre*	.523	1.44	0.35	679	315
*Acer platanoides*	.207	1.27	0.19	444	42
*Acer pseudoplatanus*	.426	4.01	1.40	906	634
*Alnus glutinosa*	.271	2.40	0.66	484	195
*Betula pendula*	.450	6.88	2.29	1,261	802
*Betula pubescens*	.596	4.09	1.09	501	127
*Carpinus betulus*	.690	3.79	1.05	755	320
*Castanea sativa*	.764	9.56	3.58	982	501
*Corylus avellana*	.344	4.47	1.47	1,282	935
*Crataegus monogyna*	.049	1.10	0.23	413	339
*Fagus sylvatica*	.496	8.45	2.91	1,388	918
*Fraxinus excelsior*	.397	4.95	1.88	1,986	1,629
*Populus tremula*	.126	NA	NA	400	16
*Prunus avium*	.589	1.98	0.56	886	401
*Prunus padus*	.004	NA	NA	394	9
*Pseudotsuga menziesii*	.596	7.66	1.96	600	193
*Quercus petraea*	.841	5.99	1.84	584	209
*Quercus robur*	.462	6.50	2.54	2,303	1,867
*Salix caprea*	.644	1.38	0.28	445	74
*Salix cinerea*	.081	0.16	0.03	392	55
*Sorbus aria*	.055	NA	NA	392	3
*Taxus baccata*	.372	2.21	0.49	518	86
*Tilia cordata*	.442	1.04	0.14	461	56
*Ulmus glabra*	.037	NA	NA	394	22
*Ulmus procera*	.013	NA	NA	393	27
