# Supplementary material for: Abundance distributions for tree species in Great Britain: A two‐stage approach to modeling abundance using species distribution modeling and random forest
Source: Ecol Evol. 2017 Jan 22;7(4):1043–56. doi: 10.1002/ece3.2661 (PMC5306018; doi:10.1002/ece3.2661)
Supplement: Supplementary file 1 [file ECE3-7-1043-s001.docx]

**Supporting information: R code walkthrough and R code**

Louise Hill

Mon Oct 03 11:55:24 2016

#############################################################################
############# CODE FOR ABUNDANCE PAPER SUPPORTING MATERIALS #################
#############################################################################

# We will produce an example map of predicted abundance of Acer campestre.
# Note that this example uses a subset of the data and models that were used
# in the published examples and so is for demonstration only.

library(biomod2)

## Loading required package: sp

## Loading required package: raster

## Loading required package: parallel

## Loading required package: reshape

## Loading required package: ggplot2

## biomod2 3.3-7 loaded.
##
## Type browseVignettes(package='biomod2') to access directly biomod2 vignettes.

library(randomForest)

## randomForest 4.6-12

## Type rfNews() to see new features/changes/bug fixes.

##
## Attaching package: 'randomForest'

## The following object is masked from 'package:ggplot2':
##
## margin

library(raster)
library(caret)

## Loading required package: lattice

###################
 ##### BIOMOD2 #####
 ###################

# Set working directory
setwd("C:/Users/user/Desktop/dummy")

# Save graphics defaults
par.defaults <- par(no.readonly=TRUE)
save(par.defaults, file="R.default.par.RData")

# Load species occupancy data
dataSpecies <- read.csv("Acer_campestre_occupancy.csv")

# Look at structure of dataSpecies dataframe
str(dataSpecies)

## 'data.frame': 18993 obs. of 4 variables:
## $ Tetrad : Factor w/ 18993 levels "HP51W","HU28A",..: 1 2 3 4 5 6 7 8 9 10 ...
## $ Easting : int 459000 421000 429000 451000 453000 321000 321000 321000 323000 323000 ...
## $ Northing: int 1213000 1181000 1183000 1181000 1189000 1001000 1003000 1005000 1003000 1005000 ...
## $ Presence: int 0 0 0 0 0 0 0 0 0 0 ...

####### Create biomod2 objects to use later #########

# Tell biomod2 which parts of the database refer to which biomod2 object
myRespName <- "Acer_campestre"
myResp <- as.numeric(dataSpecies[,"Presence"])
myRespXY <- dataSpecies[,c("Easting","Northing")]

# Load in environmental covariates
altitude <- raster("altitude")
aspect <- raster("aspect")
awctop <- raster("awctop")
bio2 <- raster("bio2")
bio4 <- raster("bio4")
bio12 <- raster("bio12")
directradiat <- raster("directradiat")
mintop <- raster("mintop")
octop <- raster("octop")
slope <- raster("slope")
soiltext <- raster("soiltext")

# Create stack of all environmental covariate layers
myExpl <- stack(list(altitude=altitude, aspect=aspect, awctop=awctop,
 bio2=bio2, bio4=bio4, bio12=bio12,
 directradiat=directradiat, mintop=mintop,
 octop=octop, slope=slope, soiltext=soiltext))

############# Choosing biomod options and running models ##############

# Create object (myBiomodData) to contain all the previous objects within it,
# formatted correctly
myBiomodData <- BIOMOD_FormatingData(resp.var = myResp,
 expl.var = myExpl,
 resp.xy = myRespXY,
 resp.name = myRespName,
 PA.nb.rep = 0)

##
## -=-=-=-=-=-=-=-=-=-=-= Acer_campestre Data Formating -=-=-=-=-=-=-=-=-=-=-=
##
## Response variable name was converted into Acer.campestre
## > No pseudo absences selection !
## ! No data has been set aside for modeling evaluation
## ! Some NAs have been automaticly removed from your data
## -=-=-=-=-=-=-=-=-=-=-=-=-=-=-=-=-= Done -=-=-=-=-=-=-=-=-=-=-=-=-=-=-=-=-=

# Set the options that you have chosen for the different model algorithms
# - here using defaults (empty arguments)
myBiomodOption <- BIOMOD_ModelingOptions()

# Run the biomod2 models that you have chosen on the data provided.
myBiomodModelOut <- BIOMOD_Modeling(
 myBiomodData,
 models = c('GLM', 'GBM', 'RF'),
 models.options = myBiomodOption,
 NbRunEval = 3,
 DataSplit = 70,
 Prevalence = 0.5,
 VarImport = 3,
 models.eval.meth = c('TSS', 'ROC'),
 SaveObj = TRUE,
 rescal.all.models = TRUE,
 do.full.models = FALSE,
 modeling.id = paste(myRespName, "Species1", sep=""))

##
##
## Loading required library...
##
## Checking Models arguments...
##
## Creating suitable Workdir...
##
## > Automatic weights creation to rise a 0.5 prevalence
##
##
## -=-=-=-=-=-=-=-=-=-= Acer.campestre Modeling Summary -=-=-=-=-=-=-=-=-=-=
##
## 11 environmental variables ( altitude aspect awctop bio2 bio4 bio12 directradiat mintop octop slope soiltext )
## Number of evaluation repetitions : 3
## Models selected : GLM GBM RF
##
## Total number of model runs : 9
##
## -=-=-=-=-=-=-=-=-=-=-=-=-=-=-=-=-=-=-=-=-=-=-=-=-=-=-=-=-=-=-=-=-=-=-=-=-=-=
##
##
## -=-=-=- Run : Acer.campestre_AllData
##
##
## -=-=-=--=-=-=- Acer.campestre_AllData_RUN1
##
## Model=GLM ( quadratic with no interaction )
## Stepwise procedure using AIC criteria
## selected formula : Acer.campestre ~ bio4 + I(altitude^2) + I(bio4^2) + I(mintop^2) +
## I(soiltext^2) + awctop + soiltext + mintop + I(bio2^2) +
## I(bio12^2) + bio2 + I(aspect^2) + aspect + I(slope^2) + slope +
## octop + I(octop^2) + I(awctop^2)
## <environment: 0x0000000020c8f798>
##
## Model scaling...
## Evaluating Model stuff...
## Evaluating Predictor Contributions...
##
## Model=Generalised Boosting Regression
## 2500 maximum different trees and 3 Fold Cross-Validation
## Model scaling...
## Evaluating Model stuff...
## Evaluating Predictor Contributions...
##
## Model=Breiman and Cutler's random forests for classification and regression
## Model scaling...
## Evaluating Model stuff...
## Evaluating Predictor Contributions...
##
##
## -=-=-=--=-=-=- Acer.campestre_AllData_RUN2
##
## Model=GLM ( quadratic with no interaction )
## Stepwise procedure using AIC criteria
## selected formula : Acer.campestre ~ bio4 + I(altitude^2) + I(mintop^2) + I(bio4^2) +
## awctop + I(soiltext^2) + soiltext + I(bio2^2) + mintop +
## I(aspect^2) + I(bio12^2) + I(awctop^2) + octop + I(octop^2) +
## I(slope^2) + slope + aspect + bio2
## <environment: 0x0000000020997fa8>
##
## Model scaling...
## Evaluating Model stuff...
## Evaluating Predictor Contributions...
##
## Model=Generalised Boosting Regression
## 2500 maximum different trees and 3 Fold Cross-Validation
## Model scaling...
## Evaluating Model stuff...
## Evaluating Predictor Contributions...
##
## Model=Breiman and Cutler's random forests for classification and regression
## Model scaling...
## Evaluating Model stuff...
## Evaluating Predictor Contributions...
##
##
## -=-=-=--=-=-=- Acer.campestre_AllData_RUN3
##
## Model=GLM ( quadratic with no interaction )
## Stepwise procedure using AIC criteria
## selected formula : Acer.campestre ~ bio4 + I(altitude^2) + I(bio4^2) + I(mintop^2) +
## I(soiltext^2) + awctop + soiltext + I(bio2^2) + mintop +
## I(bio12^2) + I(awctop^2) + I(slope^2) + slope + I(aspect^2) +
## aspect + octop + I(octop^2) + bio2
## <environment: 0x0000000020ad07e0>
##
## Model scaling...
## Evaluating Model stuff...
## Evaluating Predictor Contributions...
##
## Model=Generalised Boosting Regression
## 2500 maximum different trees and 3 Fold Cross-Validation
## Model scaling...
## Evaluating Model stuff...
## Evaluating Predictor Contributions...
##
## Model=Breiman and Cutler's random forests for classification and regression
## Model scaling...
## Evaluating Model stuff...
## Evaluating Predictor Contributions...
##
## -=-=-=-=-=-=-=-=-=-=-=-=-=-=-=-=-= Done -=-=-=-=-=-=-=-=-=-=-=-=-=-=-=-=-=

########################### Evaluating models #############################

# Get evaluation statistics for each model
myBiomodModelEval <- get_evaluations(myBiomodModelOut)

# Display the model evaluation statistics
myBiomodModelEval["TSS", "Testing.data" ,,,]

## RUN1 RUN2 RUN3
## GLM 0.630 0.649 0.632
## GBM 0.664 0.680 0.655
## RF 0.711 0.716 0.701

myBiomodModelEval["ROC", "Testing.data",,,]

## RUN1 RUN2 RUN3
## GLM 0.886 0.893 0.884
## GBM 0.907 0.911 0.903
## RF 0.925 0.927 0.923

# In this case, the Random Forest models are clearly performing the best,
# according to both model evaluation statistics. The Random Forest models
# are therefore considered to be a leading group.

# Display relative importance of each environmental covariate to each model
get_variables_importance(myBiomodModelOut)

## , , RUN1, AllData
##
## GLM GBM RF
## altitude 0.106 0.029 0.083
## aspect 0.003 0.000 0.017
## awctop 0.055 0.015 0.017
## bio2 0.013 0.014 0.093
## bio4 0.393 0.341 0.290
## bio12 0.169 0.285 0.240
## directradiat 0.000 0.000 0.021
## mintop 0.058 0.006 0.016
## octop 0.007 0.007 0.028
## slope 0.009 0.004 0.032
## soiltext 0.077 0.008 0.023
##
## , , RUN2, AllData
##
## GLM GBM RF
## altitude 0.108 0.025 0.076
## aspect 0.003 0.000 0.017
## awctop 0.056 0.015 0.016
## bio2 0.012 0.013 0.088
## bio4 0.379 0.309 0.279
## bio12 0.166 0.315 0.253
## directradiat 0.000 0.000 0.019
## mintop 0.061 0.009 0.020
## octop 0.009 0.007 0.027
## slope 0.008 0.003 0.033
## soiltext 0.066 0.005 0.019
##
## , , RUN3, AllData
##
## GLM GBM RF
## altitude 0.112 0.032 0.080
## aspect 0.003 0.000 0.015
## awctop 0.049 0.014 0.017
## bio2 0.012 0.013 0.085
## bio4 0.382 0.334 0.296
## bio12 0.180 0.313 0.260
## directradiat 0.000 0.000 0.018
## mintop 0.059 0.006 0.018
## octop 0.008 0.007 0.027
## slope 0.010 0.003 0.033
## soiltext 0.068 0.005 0.018

# Inspect model response curves for implausible responses
myGLMModels <- BIOMOD_LoadModels(myBiomodModelOut, models=c('GLM'))

myRespPlotGLM <- response.plot2(models = myGLMModels,
 Data = get_formal_data(myBiomodModelOut,'expl.var'),
 show.variables= get_formal_data(myBiomodModelOut,'expl.var.names'),
 do.bivariate = FALSE,
 fixed.var.metric = 'median',
 col = c("red", "blue", "green"),
 legend = TRUE,
 data_species = get_formal_data(myBiomodModelOut,'resp.var'))


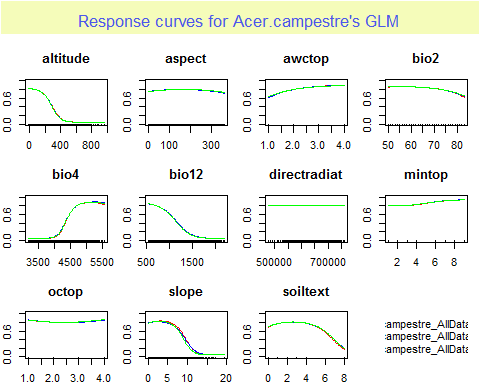


myRFModels <- BIOMOD_LoadModels(myBiomodModelOut, models=c('RF'))

myRespPlotRF <- response.plot2(models = myRFModels,
 Data = get_formal_data(myBiomodModelOut,'expl.var'),
 show.variables= get_formal_data(myBiomodModelOut,'expl.var.names'),
 do.bivariate = FALSE,
 fixed.var.metric = 'median',
 col = c("red", "blue", "green"),
 legend = TRUE,
 data_species = get_formal_data(myBiomodModelOut,'resp.var'))


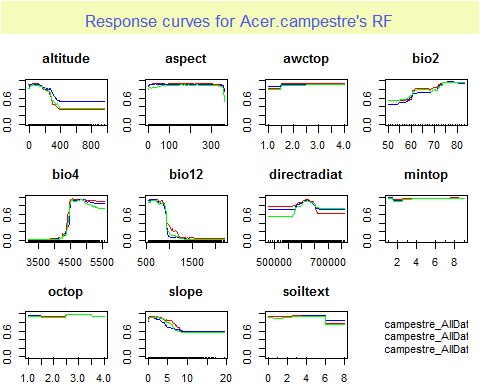


myGBMModels <- BIOMOD_LoadModels(myBiomodModelOut, models=c('GBM'))

myRespPlotGBM <- response.plot2(models = myGBMModels,
 Data = get_formal_data(myBiomodModelOut,'expl.var'),
 show.variables= get_formal_data(myBiomodModelOut,'expl.var.names'),
 do.bivariate = FALSE,
 fixed.var.metric = 'median',
 col = c("red", "blue", "green"),
 legend = TRUE,
 data_species = get_formal_data(myBiomodModelOut,'resp.var'))


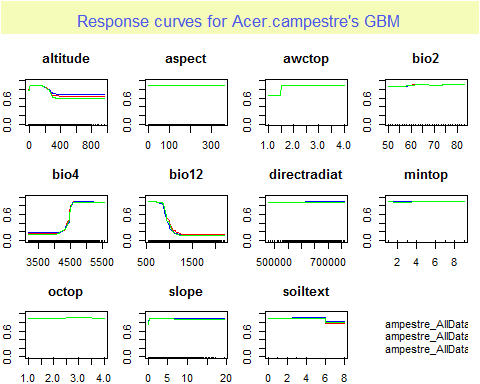


# All random forest response curves are biologically plausible, therefore
# we will use all ranodom forest models to build the ensemble model.

######################### Projecting models ###############################

# Project selected models for later building ensemble model
myBiomodProj <- BIOMOD_Projection(
 modeling.output = myBiomodModelOut,
 new.env = myExpl,
 proj.name = 'current',
 selected.models = c("Acer.campestre_AllData_RUN1_RF",
 "Acer.campestre_AllData_RUN2_RF",
 "Acer.campestre_AllData_RUN3_RF"),
 binary.meth = 'TSS',
 compress = 'xz',
 build.clamping.mask = FALSE,
 output.format = '.grd')

##
## -=-=-=-=-=-=-=-=-=-=-=-=-= Do Models Projections -=-=-=-=-=-=-=-=-=-=-=-=-=
##
## > Projecting Acer.campestre_AllData_RUN1_RF ...
## > Projecting Acer.campestre_AllData_RUN2_RF ...
## > Projecting Acer.campestre_AllData_RUN3_RF ...
##
## > Building TSS binaries
## -=-=-=-=-=-=-=-=-=-=-=-=-=-=-=-=-= Done -=-=-=-=-=-=-=-=-=-=-=-=-=-=-=-=-=

# Uncomment to plot selected models, if you want
# plot(myBiomodProj, str.grep = "RF")

# Build ensemble model from selected models.
myBiomodEM <- BIOMOD_EnsembleModeling( modeling.output = myBiomodModelOut,
 chosen.models = c("Acer.campestre_AllData_RUN1_RF",
 "Acer.campestre_AllData_RUN2_RF",
 "Acer.campestre_AllData_RUN3_RF"),
 em.by = 'all',
 eval.metric = c('ROC'),
 eval.metric.quality.threshold = c(0.6),
 prob.mean = TRUE,
 prob.cv = FALSE,
 prob.ci = FALSE,
 prob.ci.alpha = 0.05,
 prob.median = FALSE,
 committee.averaging = FALSE,
 prob.mean.weight = TRUE,
 prob.mean.weight.decay = 'proportional')

##
## -=-=-=-=-=-=-=-=-=-=-=-=-= Build Ensemble Models -=-=-=-=-=-=-=-=-=-=-=-=-=
##
## > Evaluation & Weighting methods summary :
## ROC over 0.6
##
##
## > mergedAlgo_mergedRun_mergedData ensemble modeling
## ! Models projections for whole zonation required...
## > Projecting Acer.campestre_AllData_RUN1_RF ...
## > Projecting Acer.campestre_AllData_RUN2_RF ...
## > Projecting Acer.campestre_AllData_RUN3_RF ...
##
## > Mean of probabilities...
## Evaluating Model stuff...
## > Prababilities wegthing mean...
## original models scores = 0.925 0.927 0.923
## final models weights = 0.333 0.334 0.333
## Evaluating Model stuff...
## -=-=-=-=-=-=-=-=-=-=-=-=-=-=-=-=-= Done -=-=-=-=-=-=-=-=-=-=-=-=-=-=-=-=-=

# Project and plot predictions from the ensemble model
EMplot <- BIOMOD_EnsembleForecasting(projection.output = myBiomodProj,
 EM.output = myBiomodEM)

##
## -=-=-=-=-=-=-=-=-=-=-= Do Ensemble Models Projections -=-=-=-=-=-=-=-=-=-=-=
##
##
## > Projecting Acer.campestre_EMmeanByROC_mergedAlgo_mergedRun_mergedData ...
##
## > Projecting Acer.campestre_EMwmeanByROC_mergedAlgo_mergedRun_mergedData ...
##
## -=-=-=-=-=-=-=-=-=-=-=-=-=-=-=-=-= Done -=-=-=-=-=-=-=-=-=-=-=-=-=-=-=-=-=

plot(EMplot)


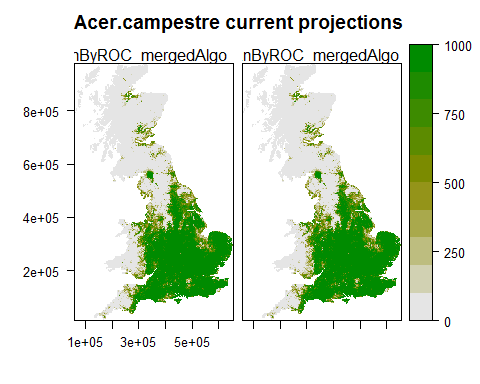


# Uncomment to save map as a raster, if you want
# Ensemble_raster <- raster("Acer.campestre/proj_current/proj_current_Acer.campestre_ensemble.grd")
# writeRaster(Ensemble_raster, file="Acer.campestre/proj_current/Acer_campestre_ensemble", format="ascii")

 ###############################
 ##### MODELLING ABUNDANCE #####
 ###############################

# load in abundance data
abundance <- read.csv("Acer_campestre_abundance.csv")

# Explore the data
str(abundance)

## 'data.frame': 287 obs. of 27 variables:
## $ AcaPp : int 957 885 920 905 905 905 920 955 950 959 ...
## $ woodarea.percent.cover : int 18 0 30 29 71 71 55 33 35 10 ...
## $ abundance.percent.cover: num 0.9 0 6 11.6 3.55 3.55 8.25 1.65 3.5 1 ...
## $ AglPp : int 377 841 952 352 649 649 939 957 457 311 ...
## $ AplPp : int 762 422 738 432 461 461 718 266 748 734 ...
## $ ApsPp : int 874 852 740 760 765 765 699 887 884 894 ...
## $ BpePp : int 719 895 986 686 572 572 986 937 690 610 ...
## $ BpuPp : int 154 47 976 325 61 61 987 199 148 52 ...
## $ CavPp : int 814 943 934 658 874 874 932 954 776 823 ...
## $ CbePp : int 793 463 799 478 355 355 799 476 681 671 ...
## $ CmoPp : int 931 935 963 949 946 946 963 957 939 944 ...
## $ CsaPp : int 308 271 994 712 366 366 981 911 244 264 ...
## $ FexPp : int 923 842 804 754 741 741 803 937 924 918 ...
## $ FsyPp : int 663 783 919 718 691 691 922 877 613 681 ...
## $ PavPp : int 697 695 944 793 500 500 937 948 486 643 ...
## $ PmePp : int 303 217 249 407 211 211 306 314 248 179 ...
## $ PpaPp : int 262 199 129 138 162 162 130 158 191 211 ...
## $ PtrPp : int 303 547 936 445 329 329 936 501 470 405 ...
## $ QpePp : int 87 93 456 180 39 39 501 746 145 250 ...
## $ QroPp : int 881 904 955 922 914 914 955 962 888 896 ...
## $ SarPp : int 540 275 385 485 273 273 408 122 452 342 ...
## $ ScaPp : int 660 707 959 420 784 784 959 801 513 593 ...
## $ SciPp : int 662 826 948 597 726 726 945 910 352 514 ...
## $ TbaPp : int 805 825 949 705 731 731 948 921 725 672 ...
## $ TcoPp : int 421 431 185 271 478 478 244 444 396 555 ...
## $ UglPp : int 865 749 267 600 745 745 106 987 763 783 ...
## $ UprPp : int 959 973 936 946 809 809 791 657 955 979 ...

head(abundance)

## AcaPp woodarea.percent.cover abundance.percent.cover AglPp AplPp ApsPp
## 1 957 18 0.90 377 762 874
## 2 885 0 0.00 841 422 852
## 3 920 30 6.00 952 738 740
## 4 905 29 11.60 352 432 760
## 5 905 71 3.55 649 461 765
## 6 905 71 3.55 649 461 765
## BpePp BpuPp CavPp CbePp CmoPp CsaPp FexPp FsyPp PavPp PmePp PpaPp PtrPp
## 1 719 154 814 793 931 308 923 663 697 303 262 303
## 2 895 47 943 463 935 271 842 783 695 217 199 547
## 3 986 976 934 799 963 994 804 919 944 249 129 936
## 4 686 325 658 478 949 712 754 718 793 407 138 445
## 5 572 61 874 355 946 366 741 691 500 211 162 329
## 6 572 61 874 355 946 366 741 691 500 211 162 329
## QpePp QroPp SarPp ScaPp SciPp TbaPp TcoPp UglPp UprPp
## 1 87 881 540 660 662 805 421 865 959
## 2 93 904 275 707 826 825 431 749 973
## 3 456 955 385 959 948 949 185 267 936
## 4 180 922 485 420 597 705 271 600 946
## 5 39 914 273 784 726 731 478 745 809
## 6 39 914 273 784 726 731 478 745 809

# Each row represents a geographical location, a 1km square.
# abundance.percent.cover is abundance of Acer campestre in hectares covered
# in each 1km square.
# For each location for which we have abundance data, the predicted
# probability of presence from the ensemble model has been extracted (this
# is the column AcaPp).
# The other columns ending Pp are the ensemble model predictions for all
# other species that we made ensemble models for, which we will also include
# in the random forest regression as variables.
# woodarea is percentage cover by woodland over 0.5ha in size within the 1km
# square (from the UK National Forest Inventory).

# Define training control: 10 fold cross-validation.
train_control <- trainControl(method="cv", number=10)

# Train the model using randomForest (rf).
model <- train(abundance.percent.cover~., data=abundance, trControl=train_control, method="rf", importance=T)

# View attributes contained in model object
attributes(model)

## $names
## [1] "method" "modelInfo" "modelType" "results"
## [5] "pred" "bestTune" "call" "dots"
## [9] "metric" "control" "finalModel" "preProcess"
## [13] "trainingData" "resample" "resampledCM" "perfNames"
## [17] "maximize" "yLimits" "times" "levels"
## [21] "terms" "coefnames" "xlevels"
##
## $class
## [1] "train" "train.formula"

# View model diagnostic information: Are we using a sufficient number of
# trees?

# Reset graphics window
load("R.default.par.RData")
par(par.defaults)

plot(model$finalModel)


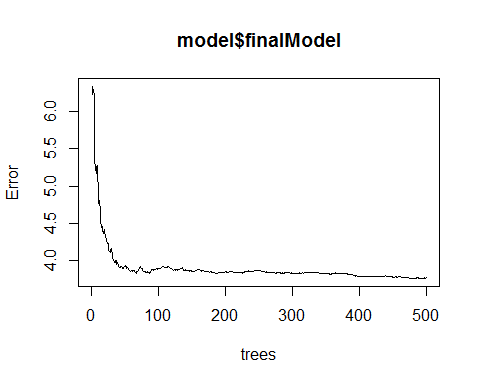


# Yes, the error rate has levelled off stabilised by 500 trees.

# The printed summary shows the R squared and Root Mean Squared Error (RMSE)
# scores, sample sizes used, the best model selected and other information.
print(model)

## Random Forest
##
## 287 samples
## 26 predictor
##
## No pre-processing
## Resampling: Cross-Validated (10 fold)
## Summary of sample sizes: 260, 257, 258, 258, 258, 259, ...
## Resampling results across tuning parameters:
##
## mtry RMSE Rsquared
## 2 1.908893 0.5084461
## 14 1.841549 0.5258427
## 26 1.846352 0.5246131
##
## RMSE was used to select the optimal model using the smallest value.
## The final value used for the model was mtry = 14.

# Make predictions and produce Mean Absolute Error (MAE) scores to evaluate
# model performance
predictions <- predict(model, abundance)
result <- data.frame(Actual=abundance$abundance.percent.cover,Predicted=predictions)
result$Difference <- abs(result$Actual - result$Predicted)
summary(result$Difference)

## Min. 1st Qu. Median Mean 3rd Qu. Max.
## 0.00062 0.09514 0.28250 0.55880 0.60750 10.13000

# Mean is MAE score

# Plot predicted vs observed abundance to inspect model performance
abundance$predictions <- predictions

predictionsFigure <- ggplot(abundance, aes(predictions, abundance.percent.cover))+
 geom_point(colour = "black")+
 labs(x = "Predicted abundance (% cover)", y = "Observed abundance (% cover)")+
 ggtitle(bquote(~italic("Acer campestre")))+
 geom_abline(slope = 1, intercept = 0)+
 theme_bw()+
 theme(aspect.ratio=1)+
 theme(plot.title=element_text(size=20, vjust=0),
 axis.text.x=element_text(angle=50, size=10, vjust=1, hjust=1),
 axis.text.y=element_text(size=10))
predictionsFigure


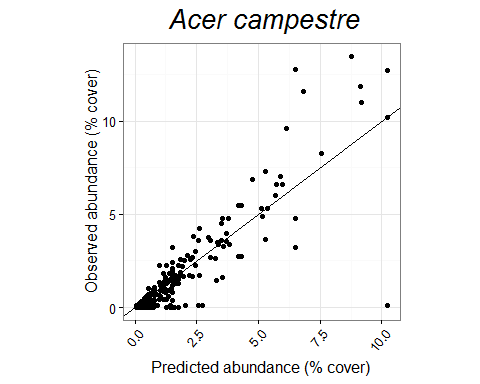


# Observed vs predicted abundance shows a strong correlation and low RMSE
# and MAE scores confirm the model is predicting the abundance of Acer
# campestre well.

# View variables by relative importance in the model to see what factors
# may be most ecologically important for this species.
plot(varImp(model))


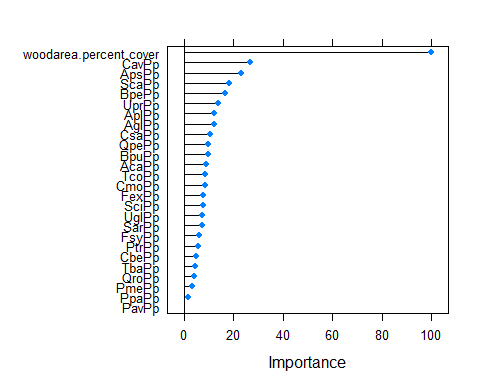


# Load dataset for predicting - this has all variables used in the random
# forest regression for the entire area we want to predict abundance to
# (whole UK - each row represents a single 1km square)
dfPrediction <- read.csv("Acer_campestre_predict.csv")
str(dfPrediction)

## 'data.frame': 220088 obs. of 26 variables:
## $ AcaPp : int 21 24 21 21 16 23 20 22 22 58 ...
## $ AplPp : int 98 140 117 109 71 167 105 131 217 383 ...
## $ ApsPp : int 121 118 121 125 111 115 119 127 129 409 ...
## $ AglPp : int 89 97 148 108 460 96 57 196 233 114 ...
## $ BpePp : int 0 6 1 1 1 2 0 2 3 34 ...
## $ BpuPp : int 408 411 421 466 748 418 354 566 561 463 ...
## $ CbePp : int 74 87 80 78 72 88 71 83 80 244 ...
## $ CsaPp : int 15 102 23 27 14 97 27 51 47 348 ...
## $ CavPp : int 43 57 77 58 189 63 39 55 77 69 ...
## $ CmoPp : int 29 95 36 51 27 122 43 108 91 311 ...
## $ FsyPp : int 44 51 41 43 56 45 44 49 47 355 ...
## $ FexPp : int 36 38 36 37 36 38 36 39 38 166 ...
## $ PtrPp : int 551 510 561 492 742 467 449 600 535 474 ...
## $ PavPp : int 1 7 1 1 11 4 1 3 3 104 ...
## $ PpaPp : int 245 357 323 288 258 451 341 444 444 514 ...
## $ PmePp : int 142 170 183 188 104 225 169 264 275 391 ...
## $ QpePp : int 342 38 639 551 397 83 507 4 10 143 ...
## $ QroPp : int 6 15 7 6 6 10 7 7 10 19 ...
## $ ScaPp : int 11 16 11 12 25 18 12 16 16 75 ...
## $ SciPp : int 43 56 38 66 44 38 40 67 61 60 ...
## $ SarPp : int 154 173 140 150 71 187 149 158 192 466 ...
## $ TbaPp : int 95 108 92 96 66 94 91 106 98 275 ...
## $ TcoPp : int 94 103 86 83 63 96 72 86 81 304 ...
## $ UglPp : int 60 81 64 54 93 47 49 72 358 632 ...
## $ UprPp : int 14 16 13 12 9 11 11 12 64 129 ...
## $ woodarea.percent.cover: int 0 0 0 0 0 0 0 0 0 0 ...

# Use RF model to predict Acer campestre abundance for all rows in the
# dataset for predicting
PredictedAbundance <- predict(model, dfPrediction, type="raw")

# Load dataset that contains the coordinates of each 1km square
ForMapping <- read.csv("Locations.csv")
str(ForMapping)

## 'data.frame': 220088 obs. of 2 variables:
## $ X: int 319500 320500 319500 320500 225500 318500 319500 320500 321500 322500 ...
## $ Y: int 975500 975500 974500 974500 973500 973500 973500 973500 973500 973500 ...

length(PredictedAbundance) == length(ForMapping$X)

## [1] TRUE

# Add predicted abundance as column to Locations file
ForMapping$PredictedAbundance <- PredictedAbundance

# Coerce to SpatialPixelsDataFrame
coordinates(ForMapping) <- ~ X + Y
gridded(ForMapping) <- TRUE

## Warning in points2grid(points, tolerance, round): grid has empty column/
## rows in dimension 1

# Coerce to raster
mapAcampestre <- raster(ForMapping)

# Set CRS for the new raster (in this case, British National Grid)
crs(mapAcampestre) <- '+proj=tmerc +lat_0=49 +lon_0=-2 +k=0.9996012717 +x_0=400000
+y_0=-100000 +ellps=airy +datum=OSGB36 +units=m +no_defs'

# Plot predicted abundance for Acer campestre
plot(mapAcampestre)


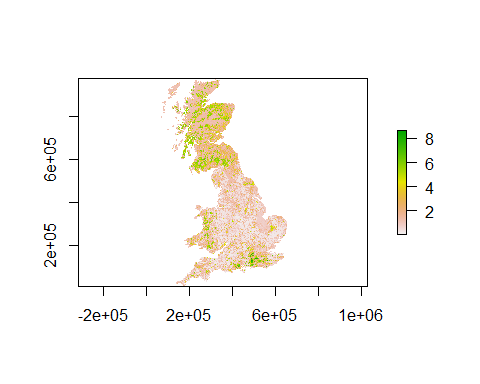


# This produces the final raster of predicted abundance of Acer campestre
# in GB. Note that this example uses a subset of the data and models that
# were used in the published examples and so is for demonstration only.

# Uncomment to save final raster
# writeRaster(mapAcampestre, "AcampestrePredictedAbundance.grd")

############################################################################
################################ END #######################################
############################################################################

**R code:**

#############################################################################

############### CODE FOR ABUNDANCE PAPER SUPPORTING MATERIALS ###############

#############################################################################

# We will produce an example map of predicted abundance of Acer campestre.

# Note that this example uses a subset of the data and models that were used

# in the published examples and so is for demonstration only.

library(biomod2)

library(randomForest)

library(raster)

library(caret)

###################

##### BIOMOD2 #####

###################

# Set working directory

setwd("C:/Users/user/Desktop/dummy")

# Save graphics defaults

par.defaults <- par(no.readonly=TRUE)

save(par.defaults, file="R.default.par.RData")

# Load species occupancy data

dataSpecies <- read.csv("Acer_campestre_occupancy.csv")

# Look at structure of dataSpecies dataframe

str(dataSpecies)

####### Create biomod2 objects to use later #########

# Tell biomod2 which parts of the database refer to which biomod2 object

myRespName <- "Acer_campestre"

myResp <- as.numeric(dataSpecies[,"Presence"])

myRespXY <- dataSpecies[,c("Easting","Northing")]

# Load in environmental covariates

altitude <- raster("altitude")

aspect <- raster("aspect")

awctop <- raster("awctop")

bio2 <- raster("bio2")

bio4 <- raster("bio4")

bio12 <- raster("bio12")

directradiat <- raster("directradiat")

mintop <- raster("mintop")

octop <- raster("octop")

slope <- raster("slope")

soiltext <- raster("soiltext")

# Create stack of all environmental covariate layers

myExpl <- stack(list(altitude=altitude, aspect=aspect, awctop=awctop,

bio2=bio2, bio4=bio4, bio12=bio12,

directradiat=directradiat, mintop=mintop,

octop=octop, slope=slope, soiltext=soiltext))

############# Choosing biomod options and running models ##############

# Create object (myBiomodData) to contain all the previous objects within it,

# formatted correctly

myBiomodData <- BIOMOD_FormatingData(resp.var = myResp,

expl.var = myExpl,

resp.xy = myRespXY,

resp.name = myRespName,

PA.nb.rep = 0)

# Set the options that you have chosen for the different model algorithms

# - here using defaults (empty arguments)

myBiomodOption <- BIOMOD_ModelingOptions()

# Run the biomod2 models that you have chosen on the data provided.

myBiomodModelOut <- BIOMOD_Modeling(

myBiomodData,

models = c('GLM', 'GBM', 'RF'),

models.options = myBiomodOption,

NbRunEval = 3,

DataSplit = 70,

Prevalence = 0.5,

VarImport = 3,

models.eval.meth = c('TSS', 'ROC'),

SaveObj = TRUE,

rescal.all.models = TRUE,

do.full.models = FALSE,

modeling.id = paste(myRespName, "Species1", sep=""))

########################### Evaluating models #############################

# Get evaluation statistics for each model

myBiomodModelEval <- get_evaluations(myBiomodModelOut)

# Display the model evaluation statistics

myBiomodModelEval["TSS", "Testing.data" ,,,]

myBiomodModelEval["ROC", "Testing.data",,,]

# In this case, the Random Forest models are clearly performing the best,

# according to both model evaluation statistics. The Random Forest models

# are therefore considered to be a leading group.

# Display relative importance of each environmental covariate to each model

get_variables_importance(myBiomodModelOut)

# Inspect model response curves for implausible responses

myGLMModels <- BIOMOD_LoadModels(myBiomodModelOut, models=c('GLM'))

myRespPlotGLM <- response.plot2(models = myGLMModels,

Data = get_formal_data(myBiomodModelOut,'expl.var'),

show.variables= get_formal_data(myBiomodModelOut,'expl.var.names'),

do.bivariate = FALSE,

fixed.var.metric = 'median',

col = c("red", "blue", "green"),

legend = TRUE,

data_species = get_formal_data(myBiomodModelOut,'resp.var'))

myRFModels <- BIOMOD_LoadModels(myBiomodModelOut, models=c('RF'))

myRespPlotRF <- response.plot2(models = myRFModels,

Data = get_formal_data(myBiomodModelOut,'expl.var'),

show.variables= get_formal_data(myBiomodModelOut,'expl.var.names'),

do.bivariate = FALSE,

fixed.var.metric = 'median',

col = c("red", "blue", "green"),

legend = TRUE,

data_species = get_formal_data(myBiomodModelOut,'resp.var'))

myGBMModels <- BIOMOD_LoadModels(myBiomodModelOut, models=c('GBM'))

myRespPlotGBM <- response.plot2(models = myGBMModels,

Data = get_formal_data(myBiomodModelOut,'expl.var'),

show.variables= get_formal_data(myBiomodModelOut,'expl.var.names'),

do.bivariate = FALSE,

fixed.var.metric = 'median',

col = c("red", "blue", "green"),

legend = TRUE,

data_species = get_formal_data(myBiomodModelOut,'resp.var'))

# All random forest response curves are biologically plausible, therefore

# we will use all ranodom forest models to build the ensemble model.

######################### Projecting models ###############################

# Project selected models for later building ensemble model

myBiomodProj <- BIOMOD_Projection(

modeling.output = myBiomodModelOut,

new.env = myExpl,

proj.name = 'current',

selected.models = c("Acer.campestre_AllData_RUN1_RF",

"Acer.campestre_AllData_RUN2_RF",

"Acer.campestre_AllData_RUN3_RF"),

binary.meth = 'TSS',

compress = 'xz',

build.clamping.mask = FALSE,

output.format = '.grd')

# Uncomment to plot selected models, if you want

# plot(myBiomodProj, str.grep = "RF")

# Build ensemble model from selected models.

myBiomodEM <- BIOMOD_EnsembleModeling( modeling.output = myBiomodModelOut,

chosen.models = c("Acer.campestre_AllData_RUN1_RF",

"Acer.campestre_AllData_RUN2_RF",

"Acer.campestre_AllData_RUN3_RF"),

em.by = 'all',

eval.metric = c('ROC'),

eval.metric.quality.threshold = c(0.6),

prob.mean = TRUE,

prob.cv = FALSE,

prob.ci = FALSE,

prob.ci.alpha = 0.05,

prob.median = FALSE,

committee.averaging = FALSE,

prob.mean.weight = TRUE,

prob.mean.weight.decay = 'proportional')

# Project and plot predictions from the ensemble model

EMplot <- BIOMOD_EnsembleForecasting(projection.output = myBiomodProj,

EM.output = myBiomodEM)

plot(EMplot)

# Uncomment to save map as a raster, if you want

# Ensemble_raster <- raster("Acer.campestre/proj_current/proj_current_Acer.campestre_ensemble.grd")

# writeRaster(Ensemble_raster, file="Acer.campestre/proj_current/Acer_campestre_ensemble", format="ascii")

###############################

##### MODELLING ABUNDANCE #####

###############################

# Load in abundance data

abundance <- read.csv("Acer_campestre_abundance.csv")

# Explore the data

str(abundance)

head(abundance)

# Each row represents a geographical location, a 1km square.

# abundance.percent.cover is abundance of Acer campestre in hectares covered

# in each 1km square.

# For each location for which we have abundance data, the predicted

# probability of presence from the ensemble model has been extracted (this

# is the column AcaPp).

# The other columns ending Pp are the ensemble model predictions for all

# other species that we made ensemble models for, which we will also include

# in the random forest regression as variables.

# woodarea is percentage cover by woodland over 0.5ha in size within the 1km

# square (from the UK National Forest Inventory).

# Define training control: 10 fold cross-validation.

train_control <- trainControl(method="cv", number=10)

# Train the model using randomForest (rf).

model <- train(abundance.percent.cover~., data=abundance, trControl=train_control, method="rf", importance=T)

# View attributes contained in model object

attributes(model)

# View model diagnostic information: Are we using a sufficient number of

# trees?

# Reset graphics window

load("R.default.par.RData")

par(par.defaults)

plot(model$finalModel)

# Yes, the error rate has levelled off stabilised by 500 trees.

# The printed summary shows the R squared and Root Mean Squared Error (RMSE)

# scores, sample sizes used, the best model selected and other information.

print(model)

# Make predictions and produce Mean Absolute Error (MAE) scores to evaluate

# model performance

predictions <- predict(model, abundance)

result <- data.frame(Actual=abundance$abundance.percent.cover,Predicted=predictions)

result$Difference <- abs(result$Actual - result$Predicted)

summary(result$Difference)

# Mean is MAE score

# Plot predicted vs observed abundance to inspect model performance

abundance$predictions <- predictions

predictionsFigure <- ggplot(abundance, aes(predictions, abundance.percent.cover))+

geom_point(colour = "black")+

labs(x = "Predicted abundance (% cover)", y = "Observed abundance (% cover)")+

ggtitle(bquote(~italic("Acer campestre")))+

geom_abline(slope = 1, intercept = 0)+

theme_bw()+

theme(aspect.ratio=1)+

theme(plot.title=element_text(size=20, vjust=0),

axis.text.x=element_text(angle=50, size=10, vjust=1, hjust=1),

axis.text.y=element_text(size=10))

predictionsFigure

# Observed vs predicted abundance shows a strong correlation and low RMSE

# and MAE scores confirm the model is predicting the abundance of Acer

# campestre well.

# View variables by relative importance in the model to see what factors

# may be most ecologically important for this species.

plot(varImp(model))

# Load dataset for predicting - this has all variables used in the random

# forest regression for the entire area we want to predict abundance to

# (whole UK - each row represents a single 1km square)

dfPrediction <- read.csv("Acer_campestre_predict.csv")

str(dfPrediction)

# Use RF model to predict Acer campestre abundance for all rows in the

# dataset for predicting

PredictedAbundance <- predict(model, dfPrediction, type="raw")

# Load dataset that contains the coordinates of each 1km square

ForMapping <- read.csv("Locations.csv")

str(ForMapping)

length(PredictedAbundance) == length(ForMapping$X)

# Add predicted abundance as column to Locations file

ForMapping$PredictedAbundance <- PredictedAbundance

# Coerce to SpatialPixelsDataFrame

coordinates(ForMapping) <- ~ X + Y

gridded(ForMapping) <- TRUE

# coerce to raster

mapAcampestre <- raster(ForMapping)

# Set CRS for the new raster (in this case, British National Grid)

crs(mapAcampestre) <- '+proj=tmerc +lat_0=49 +lon_0=-2 +k=0.9996012717 +x_0=400000

+y_0=-100000 +ellps=airy +datum=OSGB36 +units=m +no_defs'

# Plot predicted abundance for Acer campestre

plot(mapAcampestre)

# This produces the final raster of predicted abundance of Acer campestre

# in GB. Note that this example uses a subset of the data and models that

# were used in the published examples and so is for demonstration only.

# Uncomment to save final raster

# writeRaster(mapAcampestre, "AcampestrePredictedAbundance.grd")

############################################################################

################################ END #######################################

############################################################################
